# The Global Polio Laboratory Network as a Platform for the Viral Vaccine-Preventable and Emerging Diseases Laboratory Networks

**DOI:** 10.1093/infdis/jix092

**Published:** 2017-07-01

**Authors:** Ousmane M. Diop, Olen M. Kew, Esther M. de Gourville, Mark A. Pallansch

**Affiliations:** 1 World Health Organization, Geneva, Switzerland;; 2 Task Force for Global Health, Decatur, and; 3 Centers for Diseases Control and Prevention, Atlanta, Georgia; and; 4 Pan American Health Organization, Nassau, Bahamas

**Keywords:** Poliomyelitis, Eradication, Laboratory, Networking, Legacy

## Abstract

The Global Polio Laboratory Network (GPLN) began building in the late 1980s on a 3-tiered structure of 146 laboratories with different and complementary technical and support capacities (poliovirus isolation, molecular strain characterization including sequencing, quality assurance, and research). The purpose of this network is to provide timely and accurate laboratory results to the Global Polio Eradication Initiative. Deeply integrated with field case-based surveillance, it ultimately provides molecular epidemiological data from polioviruses used to inform programmatic and immunization activities. This network of global coverage requires substantial investments in laboratory infrastructure, equipment, supplies, reagents, quality assurance, staffing and training, often in resource-limited settings. The GPLN has not only developed country capacities, but it also serves as a model to other global laboratory networks for vaccine-preventable diseases that will endure after the polio eradication goal is achieved. Leveraging lessons learned during past 27 years, the authors discuss options for transitioning GPLN assets to support control of other viral vaccine-preventable, emerging, and reemerging diseases.

Following the launch of the Global Polio Eradication Initiative (GPEI) in 1988 [1], the World Health Organization (WHO) adopted a model to achieve global coverage of laboratory services to support surveillance for acute flaccid paralysis, based on the experience with eradication efforts in the Region of the Americas. Selected laboratories were enrolled into a collaborating network, the Global Polio Laboratory Network (GPLN) coordinated by WHO. Network membership depended on nomination by host government, on-site evaluation by WHO of physical infrastructure, availability of suitably qualified personnel, and ability to accurately implement recommended procedures for poliovirus detection and characterization. Although not every country has a GPLN laboratory, each is linked to designated laboratories where specimens could be referred for rapid testing. After the initial decade of growth linked to introduction of regional eradication goals and corresponding surveillance programs, GPLN membership has been mostly constant; currently, 146 laboratories are enrolled and originally categorized into 3 groups (Figure 1) with defined responsibilities: (1) subnational and national laboratories (n = 123), (2) regional reference laboratories (n = 17), and (3) global specialized laboratories (n = 6). 

**Figure 1.**
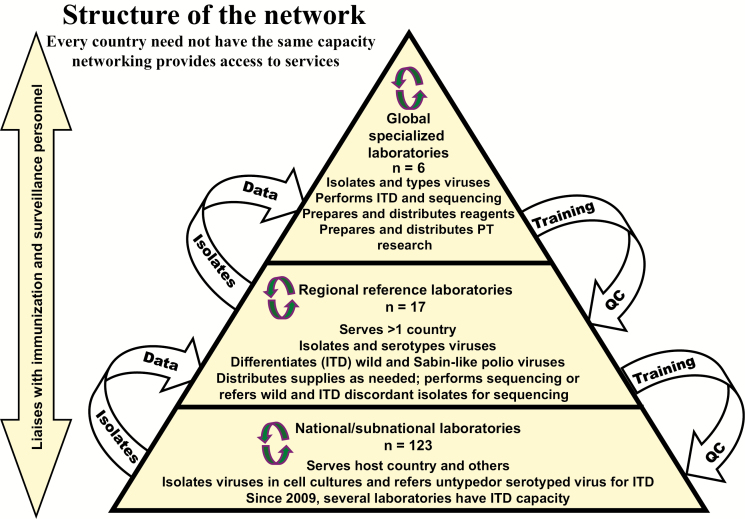
Diagram showing tiered structure and functions of the Global Polio Laboratory Network. Abbreviations: ITD, intratypic differentiation; PT, proficiency testing; QC, quality control.

Laboratory diagnosis of poliovirus consists of 3 consecutive main procedures: viral isolation for detection of the virus; intratypic differentiation to distinguish vaccine strains from wild poliovirus (WPV) strains; and, finally, sequencing for molecular epidemiology. These procedures were aligned with the 3 tiers within the GPLN. During recent years, the capacities of these laboratories have evolved tremendously, and GPLN’s laboratories have developed new capacities at all levels. It is noteworthy that >90 national/intercountry polio laboratories currently have intratypic differentiation capacity, and many regional and some national laboratories have sequencing capacities; 27 laboratories participated in the 2016 proficiency testing as part of establishing this recognized capacity. In addition, these expanded capabilities will be an important factor in fulfilling requirements of the WHO Global Action Plan to minimize the poliovirus facility–associated risk of introducing eradicated viruses into the population. As part of the process defined by this plan and with the aim of containing poliovirus in a few facilities, some specialized and regional laboratories will be designated as polio-essential facilities for containment of polioviruses.

Since the beginning of the program, a core activity of the GPLN has been to test stool specimens from patients with acute flaccid paralysis (AFP) for the presence of polioviruses. More recently, sewage specimens are also tested in several countries as a supplement to AFP surveillance. It is noteworthy that strengthening environmental surveillance is driven by the need to improve detection of poliovirus circulation in countries and areas where surveillance and immunization indicators are suboptimal. These expanded efforts were led by the GPLN and including sampling site selection, concentration of the sewage specimens, and characterization of the isolated polioviruses.

The virological data provided by the GPLN, based on confirmed wild or vaccine-derived poliovirus (VDPV) circulation, underpin the GPEI decisions regarding where targeted immunization and surveillance activities should be conducted. The data are also used to monitor progress toward polio eradication by documenting the decreasing genetic diversity and recognizing transmission links of poliovirus isolates.

Other public health programs dedicated to control or elimination of vaccine-preventable diseases (VPDs), have leveraged the GPLN model. The recently emerged or reemerged viral diseases—such as severe acute respiratory syndrome, dengue, Ebola, yellow fever, and Middle East Respiratory Syndrome coronavirus and Zika virus disease—can also benefit from GPEI assets, including the GPLN platform. As polio eradication approaches, the polio endgame opens opportunities to formulate and validate the best strategies to transition all assets generated by the GPEI. Globally coordinated political and financial efforts and integrated platforms to control, eliminate, and eradicate infectious diseases will be needed to secure polio eradication and add value to other current and future public health initiatives. However, care should be taken not to jeopardize polio eradication goals by premature transitioning.

## TECHNICAL PILLARS OF THE GPLN

### Poliovirus Detection and Characterization Procedures

WHO has standardized procedures used in the GPLN for testing of stool specimens from AFP cases. A consensus on recommended procedures was achieved through periodic informal consultative meetings convened by WHO for discussions among GPLN subject-matter experts. Briefly, stool specimens from AFP cases are inoculated into cell cultures, and any polioviruses isolated have serotype and intratype determined. Partial genomic nucleotide sequences are generated for wild and programmatically important Sabin-related poliovirus isolates, and phylogenetic relationships among isolates are analyzed to investigate transmission linkages [2, 3]. 

None of the procedures used in the GPLN are available as commercial kits. Therefore, WHO assigns responsibility for production of standardized quality assured reagents to a few global specialized laboratories that also coordinate reagent and proficiency test distribution and collaborate to train personnel. The GPLN evaluates reagents, procedures and proficiency test materials under field conditions and on different equipment platforms to document performance characteristics and resolve operational concerns, in order to ensure accuracy of testing before adoption of new procedures.

### Capacity Building

At enrollment, GPLN members varied in their capacities for laboratory testing. WHO laboratory coordinators in headquarters and all 6 regions, then and now, evaluate needs and leverage support from government and/or GPEI international partners to fill identified gaps and ensure uninterrupted availability of trained personnel; material resources for testing, analysis, and reporting of results; and appropriate infrastructure for safe storage and handling and disposal of infectious materials. A mixed approach is used to train personnel. Regional workshops are the most cost-effective way to simultaneously train multiple persons using standardized training curricula that allows time for practical work and covers theoretical aspects of procedures, equipment (selection, maintenance, and calibration), results analysis and interpretation, troubleshooting, reporting, and discussion of safety. An added benefit is the fostering of collaborative linkages with facilitators and peer learning among participants. Two other training approaches are on-site training conducted by WHO staff or consultants, and trainee assignments to reference laboratories.

### Indicators for Monitoring Laboratory Performance

Process indicators are used to monitor completion of each stage of testing and overall reporting time to ensure the timely availability of results for program action. Outcome indicators monitor the accuracy of testing through testing of quality control materials and annual proficiency tests. Overall quality assurance is monitored through annual auditing of network laboratories by WHO-assigned external reviewers, who use a standardized checklist and scoring system to determine whether laboratories meet defined performance criteria. Laboratories that fail the audit must test samples in parallel with an accredited reference laboratory until performance problems are resolved.

### Results Reporting, Data Sharing, Management, and Ownership

**Figure 2.**
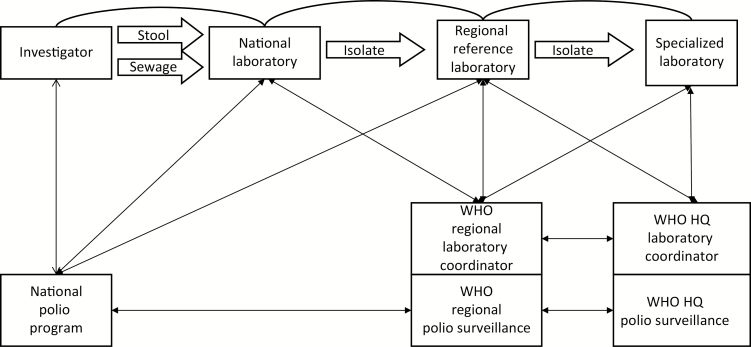
Diagram showing laboratory data flow in the Global Polio Eradication Initiative. Abbreviation: WHO HQ, World Health Organization headquarters.

The GPEI is a data-driven program. Laboratory results confirm geographic locations of poliovirus transmission and are used in planning of responsive immunization actions. Data sharing relationships were established among laboratories and immunization and surveillance personnel (Figure 2). In early phases of the GPEI, electronic data management systems and Internet were absent from many laboratories in resource-constrained settings. WHO mobilized resources and designed databases, procured computer and communication equipment, and trained laboratory personnel in electronic data management. 

Currently, laboratories share databases weekly with WHO and national authorities. WPV, VDPV, and partial genomic nucleotide sequence results, however, are shared within 1–2 days of their availability. Separate databases are maintained by laboratories that sequence viruses, but sequences are shared among laboratories as needed to perform phylogenetic analyses, particularly related to virus importations and VDPV emergence. In the event of new scientific discoveries, all laboratories making significant contributions to testing and analysis are represented among coauthors of scientific publications.

### Changes in Management

**Figure 3.**
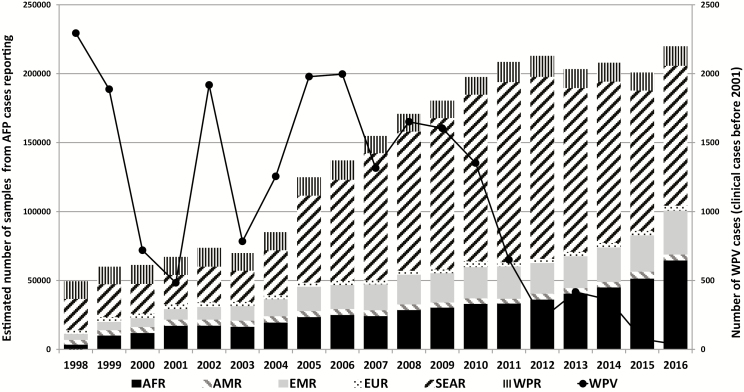
Changes in detections of acute flaccid paralysis (AFP) and wild poliovirus (WPV) between 1998 and 2016. Abbreviations: AFR, African Region; AMR, Region of the Americas; EMR, Eastern Mediterranean Region; EUR, European Region; SEAR, South-East Asia Region; WPR, Western Pacific Region.

As the GPEI program evolved, changes to AFP surveillance policies (eg, universal adoption of virological confirmation and discontinuing use of the poliomyelitis clinical case definition) and performance targets (AFP case detection rate shifted from 1 to 2 per 100000 in persons <15 years of age in polio-endemic regions) [4, 5] caused significant increases in laboratory workloads (Figure 3). The GPLN responded with diverse approaches that included increasing staffing levels and testing supplies redistribution of specimens to other laboratories with capacity, and extending work time or adding work shifts to manage workload.

**Figure 4.**
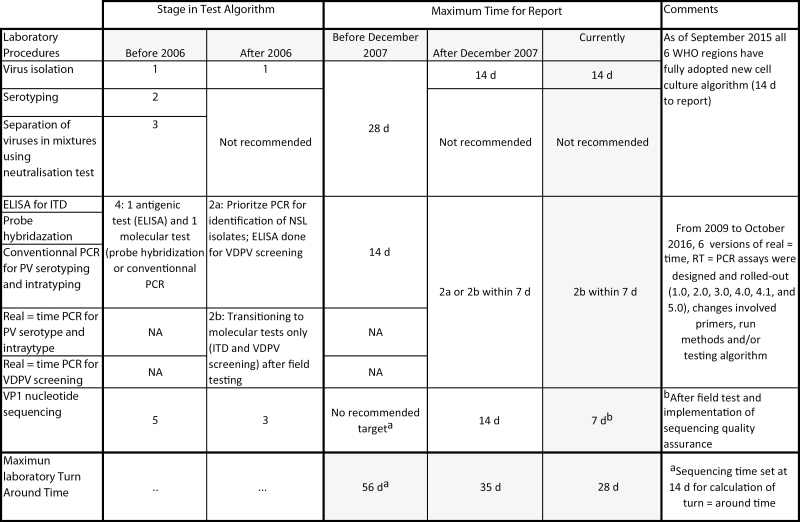
Global Polio Laboratory Network changes to testing algorithm to shorten laboratory reporting time. Abbreviations: ELISA, enzyme-linked immunosorbent assay; NA, not applicable; NSL, non–Sabin-like; PCR, polymerase chain reaction; PV, poliovirus; RT-PCR, reverse-transcription PCR; VDPV, vaccine-derived poliovirus; VP1, viral protein 1; WHO, World Health Organization.

As foci and intensity of WPV transmission decreased, WHO shortened target laboratory reporting time (Figure 4) to expedite implementation of responsive immunization campaigns. The GPLN achieved a 50% reduction in reporting time by changing the testing algorithm, which shortened the observation time for inoculated cell cultures and replaced serological based testing with faster molecular tests to determine serotype and intratype and screen for VDPVs [2, 6, 7]. Another significant change was building capacity in more laboratories to perform molecular tests, which eliminated the time associated with shipping specimens and isolates between laboratories.

Detection of a poliomyelitis outbreak in Hispaniola caused by circulation of VDPV type 1 viruses [8] caused further changes in the GPLN. Retrospective nucleotide sequencing of archived Sabin-related polioviruses confirmed a prior multiyear VDPV type 2 outbreak in Egypt. The GPLN developed, evaluated, and implemented a real-time polymerase chain reaction procedure to routinely screen Sabin-related isolates to detect certain key mutations commonly found in VDPVs [7]. During field evaluation of the method, VDPV outbreaks were retrospectively detected in the Democratic Republic of Congo, Ethiopia, and Nigeria [9].

Suspicions about weaknesses in AFP surveillance based on detection of genetic gaps among WPVs in some countries led to adoption of a supplemental surveillance approach that involves testing sewage samples collected from the environment or treatment plants [10]. Since 2000, this supplemental approach was newly established or expanded in selected countries, with results routinely used for program monitoring and planning. (Countries conducting supplementary environmental surveillance for PV detection include Afghanistan, Angola, Bangladesh, Burkina Faso, Cameroon, Chad, Egypt, Guinea, India, Indonesia, Madagascar, Niger, Nigeria, Pakistan, Senegal, and South Africa.) Looking to the future, the GPLN spearheaded policy development requiring containment of polioviruses through destruction or use of enhanced biosafety and biosecurity for handling of polioviruses in the posteradication era.

### Characterization of Costs

The GPLN surveyed its members to characterize the costs and funding sources for work done in support of the GPEI [11]. The survey documented that governments are the major funders, meeting the majority of operational and staffing costs for laboratories. GPEI partners mostly support training of personnel, production of standardized test reagents and control materials, proficiency test programs, procurement of commodities in a few reference laboratories that serve multiple countries, and personnel costs associated with network coordination. These survey results were used by WHO for planning and resource mobilization. A key finding of this survey is that approximately 10% of the GPEI’s total AFP surveillance budget is spent on the GPLN, which highlights the fact that the expected value of surveillance information from the GPLN currently exceeds its costs.

## LEVERAGING GPLN ASSETS TO SUPPORT OTHER PUBLIC HEALTH INITIATIVES

An enduring contribution of polio eradication and the GPLN is the close integration on a global scale of field and laboratory-based surveillance and public health action. Before the launch of the GPEI and development of the GPLN, such integration was sporadic in many settings and virtually nonexistent in others. The GPLN opened an avenue for virologists and microbiologists worldwide to contribute more directly to the public health needs within and beyond their own communities. The clearly evident benefits of the GPEI and other VPD control programs fostered development of additional global and Regional laboratory networks supporting surveillance for other VPDs. Conditions for establishment and maintenance of laboratory networks, however, vary widely across WHO regions and the phases of each VPD control program.

### Adding Value by Laboratory Networking

Establishment of the first comprehensive regional polio laboratory network in the Americas benefitted from the relative prosperity of the region, a widely shared Pan-American perspective, and the prospect of regional polio eradication within 5 years [12]. However, with certification of polio-free status by the Regional Certification Commission in 1994 [13], emphasis quickly shifted from polio to other VPDs, especially measles and rubella, and new regional networks grew from the polio network. This serially focused approach to VPD control aimed to leverage the gains in vaccine coverage and surveillance achieved through polio eradication, while effectively using the limited public health resources available. Success hinged on the relative geographic separation of the Americas and the moderate transmissibility of poliovirus. 

The Western Pacific Region, with China at its center, has maintained sensitive poliovirus surveillance since certification in 2000 [14], as the risk of outbreaks from imported virus [15, 16] and VDPV emergence continues [17, 18], even as it expands laboratory support to broader VPD control activities. The European Region, certified polio free in 2002 [19] has maintained high rates of vaccine coverage in Western and Central Europe, but coverage and integrated poliovirus surveillance declined as resources were redirected in some Central Asian and Eastern European countries, leading to outbreaks from imported WPV [20] and VDPVs [18]. Virologists in India and other countries in the South-East Asia Region have continued to support intensive, sensitive poliovirus surveillance since certification in 2014 [21], as their colleagues in the African and Eastern Mediterranean regions continue to track the last chains of WPV transmission [22] and alert the GPEI of the emergence of VDPVs [18]. All GPLN laboratories are needed to help the GPEI safely navigate the polio endgame [23].

Laboratories of the GPLN have been instrumental in the initial detection and characterization of newly emerging pathogens and diseases of public health importance, including severe acute respiratory syndrome, outbreaks of paralytic disease associated with enterovirus 71, and Middle East respiratory syndrome coronavirus. Many of the molecular methods first introduced by the GPLN, such as diagnostic polymerase chain reaction and genetic sequencing as an epidemiological tool, are now widely applied throughout other laboratory networks to identify and track infectious disease agents (eg, Ebola recently).

### Building on Geographic and Technical Convergences Between Laboratory Networks

From the outset, polio eradication was seen as the next step in a long-term strategy for global control of VPDs, with the concomitant strengthening of global public health infrastructure, including public health laboratories. Active collaboration across VPD laboratory networks is facilitated by overlapping technical expertise, frequent colocation of viral VPD laboratories (eg, measles/rubella, rotavirus, yellow fever [in Africa]. and Japanese encephalitis [in South-East Asia and Western Pacific regions]), shared facility resources, often under the same laboratory leadership, and the shared imperative to support infectious diseases elimination or control activities in different regions.

### Ensuring Continued Support to Public Health Activities

Although continued poliovirus surveillance and laboratory investigations remain essential to secure the endgame [23], the GPLN will continue to serve as a model for infectious disease laboratory networks. This is especially true for VPD networks, which support efforts to “go on offense” against infectious diseases. Although VPD networks have still untapped potential to support key public health initiatives, many VPD laboratories and their supporting WHO coordinators are severely overstretched. Further expansion of capacity will require additional investments in human as well as material resources, investments which have proved to be highly cost-effective. Fundamental to this goal is the need for (1) strong ownership of laboratory networks by countries and/or regional bodies, including sustained funding, and (2) investments by all countries in the academic training of laboratory staff prepared to support future, broadened public health initiatives.

## PLANNING GPLN’S TRANSITION TO SUPPORT OTHER PUBLIC HEALTH ACTIVITIES

### Situational Analysis

**Figure 5.**
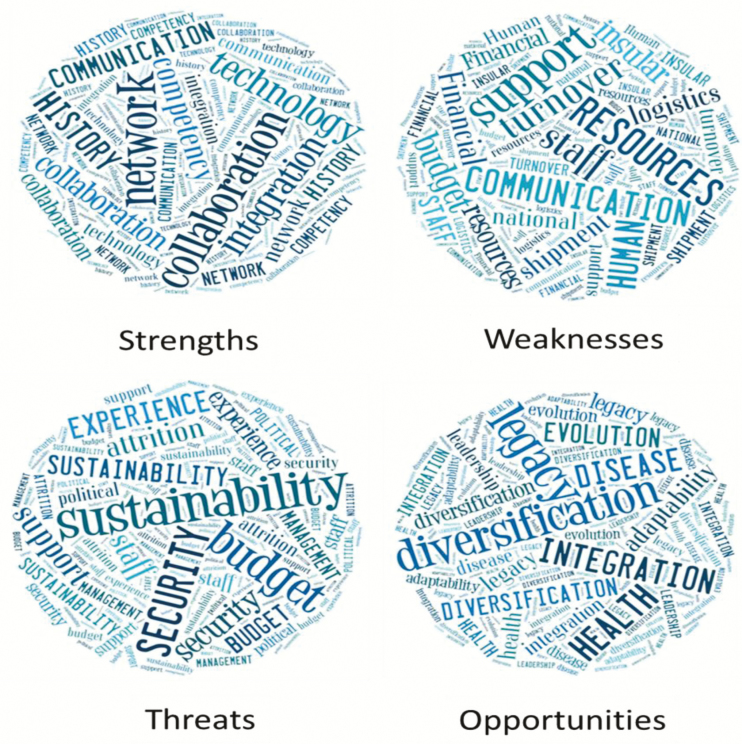
Word cloud analysis based on the frequency of words used during the SWOT (strengths, weaknesses, opportunities, and threats) analysis. When some key words were cited in different categories, their sizes are proportional to their relevance in the each categories.

A SWOT (strength, weaknesses, opportunities, and threats) analysis after 25 years of GPLN functioning was conducted in 2014. The main outcomes are compiled in Figure 5. In summary, the members of the GPLN have stressed the following strengths: the network structure, the efficient collaboration/coordination at different levels within the GPLN, the integration with the Expanded Program for Immunization, the high competency and reliability of laboratory personnel, the ability to adopt new methods, an excellent quality assurance system ensuring accuracy of results, and a strong laboratory data management system. The main areas where the GPLN should continue improvement efforts include finding sustainable mechanisms for national support, streamlining financial support to laboratories, and strengthening managerial skills at laboratory level. Lessons learned from the GPLN should help foster interregional relationships under the Global Health Security Agenda framework to build countries’ capacities and optimize costs to prevent, detect, and respond to infectious diseases threats.

Over the years, the GPLN has efficiently demonstrated capacity to overcome weaknesses and threats. As an example, communication with Expanded Program for Immunization personnel has been improved, ensuring tight and efficient links between the 2 components of the AFP surveillance system. Moreover, the high turnover rate of competent staff has always been compensated by regular training and integration of new staff.

In addition, whenever funding or capacity building opportunities were offered by GPEI partners, the GPLN was able to leverage resources and innovate to maintain high quality standards. Implementation of environmental surveillance of polioviruses in 13 GPLN laboratories in the African, Eastern Mediterranean, and South-East Asia regions during the last 5 years highlights the receptivity to changes aiming to improve diagnostic capacities of the network [24]. Rapid evolution and adoption of molecular assays for detection of WPVs and VDPVs is another significant example.

### Need for Protecting Investments and Sustaining Services Until Global Certification and Beyond

Since its creation, the GPLN has accumulated tremendous investments in human resources, infrastructures and equipment. Furthermore it was able to build an efficient networking system to proactively identify gaps and propose adequate solutions and be able to efficiently conduct troubleshooting, learn lessons and develop improvement plans. As an example, the real-time monitoring of poliovirus evolution has permitted timely adaptation of molecular diagnostic procedures in 2015 to detect viruses belonging to new clusters or lineages. At this stage of the GPEI, it is of utmost importance to protect these investments, mainly the biggest assets of the GPLN, its well-trained and dedicated human resources and efficient operational principles.

Indeed, there is a need to sustain the services and contributions of polio laboratories through and beyond the process of global certification of poliomyelitis eradication. Transitioning the GPLN assets toward other public health programs is an important part of the endgame strategic plan, and it is noteworthy that GPEI partners increasingly recognize the worth of building health systems rather than focusing on specific vertical programs or initiatives.

A quick review of the ongoing consultations and brainstorming on this area of work points toward different options for the future of the GPLN, the main ones being evolution toward regional laboratory networks devoted to specific diseases, integration with existing global VPD laboratory networks, and transforming the GPLN to a core network for broader support of public health activities and emergencies.

It is clear and consensual that an efficient public health laboratory is needed in all countries, but to reach this goal several challenges need to be overcome. First, in many low-resource countries, the health laboratory network is nonexistent owing to lack of infrastructure, which hampers adequate mainstreaming of polio surveillance functions (needed in the long-term) into national activities. Second, this heterogeneity among countries’ capacities makes difficult to align visions, secure resources, and ensure efficient in-country implementation of any transition plan. Finally, it is critical to build networking capacity up front among all stakeholders to ensure successful transition.

For each of these options it is necessary to conduct in-depth analysis of costs and comprehensively weigh health and financial benefits, cost-effectiveness, and cost savings. Indeed, maintaining political commitment and funding from countries and donors is the key driver for a successful transition of GPLN assets to support public health programs, and this can be obtained only if clear and measurable objectives are established and benefits are clearly delineated.

## KEY PREREQUISITES TO ENSURE THE SUCCESS OF THE TRANSITION

### Aligning Visions of GPEI Partners

There are several enabling factors for successful transition of GPLN assets. The first is alignment across the GPEI partnership on the direction of the program after certification, which will provide a solid ground for a platform that includes (1) retaining a postcertification surveillance capacity to sustain polio eradication, (2) continuing to share assets with established laboratory networks for effective integration, and (3) using human resources and operational assets to provide support for emerging and reemerging diseases outbreaks.

### Funding

Development of this platform can meet the interests of different stakeholders and donors to build health systems rather than funding competing health programs that affect performance of highly qualified laboratory personnel and cost-effectiveness. However, because donor funding is difficult to predict, maintaining the fundraising capacity of the GPEI should be part of the global transition plan. The model of polio surveillance to be implemented after eradication, mainly the environmental surveillance of poliovirus in this model, will be the cost driver and needs to be carefully considered to ensure the sustainability of the platform. Indeed, when eradication of polio will be achieved after several missed milestones and setbacks, engagement of different stakeholders for long-term support may change. At that point, the best insurance that base-level funds will continue to be available is to convince donors of the cost-effectiveness of their investments.

### Delineating Short- and Long-Term Transition Activities

From lessons learned in building laboratory capacity and networks, a phased transitioning strategy of GPLN assets that clearly distinguishes short-term activities after certification (section Aligning Visions of GPEI Partners above) and a long-term plan seems the best way to develop the platform successfully. While moving forward with this process, the GPLN should ensure that unfinished works in the current strategic plan are completed (eg, diversification and strengthening of laboratory personnel’s managerial skills, renewal of old equipment, and scale-up of effective modern communication and information systems).

## CONCLUSIONS

**Figure 6.**
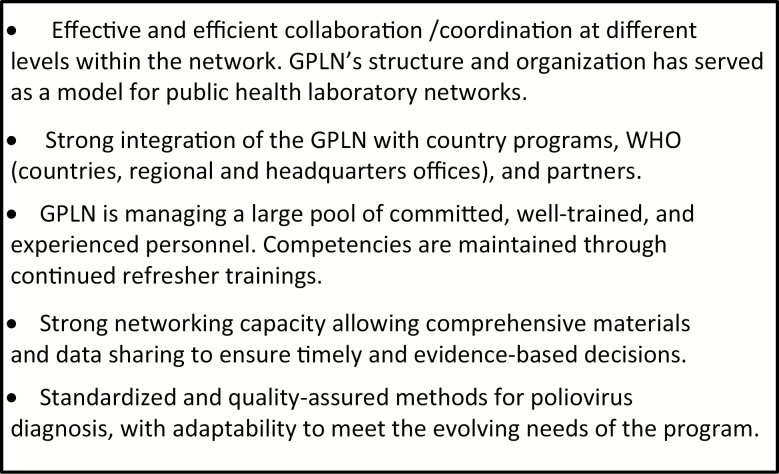
Main achievements of the Global Polio Laboratory Network (GPLN). Abbreviation: WHO, World Health Organization.

The GPLN since its creation in 1989 has provided valuable support to the GPEI by ensuring that timely and accurate data are available to orient both field surveillance and immunization activities. Although polio eradication is getting closer, it is of utmost importance to secure eradication but also plan for transitioning skills and assets that were built worldwide during >2 decades. Because the GPLN is perceived as one valuable part of the GPEI to be leveraged, this article describes some of its achievements (Figure 6) and potential avenues for better use of this network after certification. At the GPEI level, ongoing work aims to identify opportunities for integration of polio assets and/or functions into other program areas or mainstream them into the national health infrastructure.

The simplest thinking is to fully transition GPLN assets to VPD networks that have already built their capacities based on the GPLN model. In fact, integration of the GPLN with these networks is already a reality in places where both capacities coexist in the same laboratory. This integration of (1) skills and assets (human resources, infrastructures, equipment, and consumables) and (2) activities related to quality management needs to be pursued and reinforced, the prerequisite being sustained funding of the GPLN. It is not certain that the level of funding for eradication, elimination, and/or control initiatives will be maintained. Importantly, GPLN’s skills and assets that can really make the difference are the established networking system and the highly trained staff in charge of operations to ensure both the efficiency and sustainability of laboratory support to eradication programs but also to other public health interventions where and when needed (eg, influenza pandemic or Ebola crisis).

Therefore, transition planning to enable both (1) maintenance of a polio diagnostic capacity to secure polio eradication and (2) development of a core capacity/platform, which can be used to support VPDs but also outbreaks or events of emerging or reemerging diseases, will be the best trade-off to reconcile and manage expectations from GPEI’s partners, other global public health initiatives, and WHO member states. Furthermore, this process will allow retention of skilled laboratory staff by engaging them in new areas of work, preserve the essential linkages between field and laboratory surveillance, and consequently maintain the capacities of countries to respond to health emergencies.

While planning the transition of GPLN assets and skills toward support of other public health initiatives, it is important to comprehensively map out, evaluate, and document investments, assets, and skills that have maintained and strengthened the GPLN system, including networking capacity, for nearly 27 years. This information will inform a GPEI position paper on how to manage risks and opportunities associated with the eradication of polio and provide support to member states for a successful transition.

## Notes

***Acknowledgments.*** We would like to express strong appreciation for the invaluable work done by all past and current WHO Polio Laboratory Network Coordinators (Humayun Asghar, Charles Byabamazima, Annick Dosseh, Evgeniy Gavrilin, Nicksy Gumede-Moeletsi, Fem Paladin, Sirima Pattamadilok, Gloria Rey Benito, and Yan Zhang) to maintain high performance standards within the GPLN. We are also grateful to laboratory scientists and other workers of the 146 laboratories of the GPLN, as well as the surveillance field workers and country program counterparts at all levels working in the GPEI. Last, building and strengthening the GPLN would not be possible without the sustained contributions of the GPEI international partnership to the effort, namely, WHO, Rotary International, the Centers for Disease Control and Prevention, the United Nations Children’s Fund (UNICEF), the US Agency for International Development, and the Bill & Melinda Gates Foundation.

As the Polio Eradication Initiative is getting closer to celebrating eradication, this article emphasize achievements and reflect on transitioning assets and skills of a key part of the Program, the Global Polio laboratory Network, to support other public health initiatives.

***Supplement sponsorship.*** This work is part of a supplement coordinated by the Task Force for Global Health with funding provided by The Bill and Melinda Gates Foundation and the Centers for Disease Control and Prevention.

***Disclaimer.*** Authors are staff members of the World Health Organization, the Pan American Health Organization, the Task Force for Global Health, and the US Centers for Diseases Control and Prevention. The authors alone are responsible for the views expressed in this publication, and they do not necessarily represent the decisions, policy, or views of the World Health Organization, the Task Force for Global Health, the Pan American Health Organization, or the Centers for Diseases Control and Prevention.

***Potential conflicts of interest.*** All authors: No reported conflicts. All authors have submitted the ICMJE Form for Potential Conflicts of Interest. Conflicts that the editors consider relevant to the content of the manuscript have been disclosed.

## References

[1] World Health Organization. WHA resolution 41.28. Global polio eradication by the year 2000 http://www.who.int/ihr/polioresolution4128en.pdf Accessed 12 December 2016.

[2] World Health Organization. WHO polio laboratory manual http://whqlibdoc.who.int/hq/2004/WHO_IVB_04.10.pdf Accessed 12 December 2016.

[3] World Health Organization. Supplement polio laboratory manual http://polioeradication.org/polio-today/polio-now/surveillance-indicators/the-global-polio-laboratory-network-gpln/ Accessed 12 December 2016.

[4] World Health Organization. Field guide for supplementary activities aimed at achieving polio eradication. Publication WHO/EPI/GEN.95.1. Geneva, Switzerland: World Health Organization, 1995.

[5] World Health Organization. WHO AFP surveillance guidelines http://www.polioeradication.org/dataandmonitoring/Surveillance.aspx Accessed 12 December 2016.

[6] Kilpatrick DR Yang CF Ching K Rapid group-, serotype-, and vaccine strain-specific identification of poliovirus isolates by real-time reverse transcription-PCR using degenerate primers and probes containing deoxyinosine residues. J Clin Microbiol 2009; 47:1939–41. 19386844 10.1128/JCM.00702-09 PMC2691077

[7] Kilpatrick DR Ching K Iber J Identification of vaccine-derived polioviruses using dual-stage real-time RT-PCR. J Virol Methods 2014; 197:25–8. 24321704 10.1016/j.jviromet.2013.11.017 PMC8215842

[8] Kew O Morris-Glasgow V Landaverde M Outbreak of poliomyelitis in Hispaniola associated with circulating type 1 vaccine-derived poliovirus. Science 2002; 296:356–9. 11896235 10.1126/science.1068284

[9] World Health Organization. Fifteenth informal consultation of the Global Polio Laboratory Network (GPLN)—June 2009 http://polioeradication.org/tools-and-library/policy-reports/who-weekly-epidemiological-record/gpln-publications/ Accessed 12 December 2016.

[10] Asghar H Diop OM Weldegebriel G Environmental surveillance for polioviruses in the Global Polio Eradication Initiative. J Infect Dis 2014; 210:S294–303. 25316848 10.1093/infdis/jiu384 PMC10578309

[11] de Gourville E Duintjer Tebbens RJ Sangrujee N Pallansch MA Thompson KM Global surveillance and the value of information: the case of the global polio laboratory network. Risk Anal 2006; 26:1557–69. 17184397 10.1111/j.1539-6924.2006.00845.x

[12] Pan American Health Organization. Director announces campaign to eradicate poliomyelitis from the Americas by 1990. Bulletin PAHO 1985; 19:21–35.

[13] Robbins FC de Quadros CA Certification of the eradication of indigenous transmission of wild poliovirus in the Americas. J Infect Dis 1997; 175:S281–5. 9203731 10.1093/infdis/175.supplement_1.s281

[14] World Health Organization. Certification of poliomyelitis eradication: Western Pacific Region. Wkly Epidemiol Rec 2000; 75:399–400. 11189701

[15] World Health Organization. Wild poliovirus imported into Qinghai province, China. Wkly Epidemiol Rec 2000; 75:55–7. 11265275

[16] Luo HM Zhang Y Wang XQ Identification and control of a poliomyelitis outbreak in Xinjiang, China. N Engl J Med 2013; 369:1981–90. 24256377 10.1056/NEJMoa1303368

[17] Burns CC Diop OM Sutter RW Kew OM Vaccine-derived polioviruses. J Infect Dis 2014; 210(suppl 1):S283–93. 25316847 10.1093/infdis/jiu295

[18] Jorba J Diop OM Iber J Sutter RW Wassilak SG Burns CC Update on vaccine-derived polioviruses—worldwide, January 2015–May 2016. MMWR Morb Mortal Wkly Rep 2016; 65:763–9. 27491079 10.15585/mmwr.mm6530a3

[19] World Health Organization. Certification of poliomyelitis eradication—European Region, June 2002. Wkly Epidemiol Rec 2002; 77:221–3.12125241

[20] World Health Organization. Poliomyelitis in Tajikistan: first importation since Europe certified polio-free. Wkly Epidemiol Rec 2010; 85:157–8. 20449940

[21] Bahl S Kumar R Menabde N Polio-free certification and lessons learned—South-East Asia Region, March 2014. MMWR Morb Mortal Wkly Rep 2014; 63:941–6. 25340910 PMC5779468

[22] Snider CJ Diop OM Burns CC Tangermann RH Wassilak SG Surveillance systems to track progress toward polio eradication—worldwide, 2014–2015. MMWR Morb Mortal Wkly Rep 2016; 65:346–51. 27054558 10.15585/mmwr.mm6513a3

[23] World Health Organization. Polio Eradication and Endgame Strategic Plan 2013–2018 http://polioeradication.org/who-we-are/strategy/ Accessed 12 December 2016.

[24] World Health Organization. Polio environmental surveillance expansion plan http://polioeradication.org/wp-content/uploads/2016/07/GPLN_Expansion PlanES.pdf. Accessed 12 December 2016.

